# The Evolution of the Modern Hospital

**Published:** 1896-08-08

**Authors:** 


### The Evolution of the Modern Hospital.

### I.—THE GROWTH OF THE HOSPITAL IDEA.

Although the hospital idea in various forms has been familiar to mankind for many hundreds of years, there can be no question that by a process of evolution it has now arrived at a point of perfection, which makes a few great hospitals of recent years models of completeness. At first the idea merely expressed the kindly feeling which animated one man towards his fellow when stricken by accident or disease. In the most ancient times it was customary to carry out the sick into the market place or some other part of the town, or to place them somewhere where they were likely to be seen by the greatest number of people, in the hope that someone might be found who would be able to discover the nature of the ailment and to suggest a suitable remedy. As time went on benevolent people, or the community as represented by the constituted authorities, began to set aside a portion of some existing building for the accommodation of the sick. In this way wealthy people cared for the sick wayfarer; the bishops provided house room for the sick who were brought to their palaces; the priests, who in early days were largely the physicians of the people, provided accommodation in connection with the monasteries, just as in earlier times portions of the temples, or buildings located in the groves adjacent to the temples, were devoted to similar purposes.

In the earlier centuries of Christianity much was done to develop the hospital idea. During the middle ages that idea was largely lost sight of, to be again actively developed some two centuries ago by the erection of hospitals or hotels-dieu, which were entirely devoted to the reception and care of the sick. During the next hundred and fifty years hospitals multiplied everywhere, but their insanitary condition made them deservedly unpopular, and as a consequence no one would enter a hospital who could possibly help it. The Crimean war and the horrors attending the absence of proper care for the wounded produced Miss Nightingale, who brought about a revolution in the treatment of the sick, and introduced changes which have created a reforming movement in regard to these matters throughout the civilised world. Miss Nightingale, whose first practical knowledge was gained at Kaiserwurth—a fact which is too often forgotten—by her administrative ability and practical knowledge convinced a slumbering world of the importance of securing trained nurses for the sick. Nearly twenty years elapsed before her crusade brought conviction to hospital managers and members of the medical profession to a sufficient extent to secure the radical reforms which were called for in the management of the hospitals of this country, and subsequently of other countries, too. She, however, persevered; and by her munificence in setting aside the nation's gift to herself for the purpose of establishing and maintaining the Nightingale School for Training Nurses, ultimately secured those reforms which have since done so much to preserve human life and mitigate human suffering.

It is interesting to note that Sir Joseph Lister was passing through the medical schools and qualifying for the practice of his profession about the time when Miss Nightingale was training as a nurse, and then giving her object-lesson in improved methods in the care of the sick in hospitals in the Crimea. Three years after peace had been declared, Sir Joseph Lister published his paper on "Early Stages of Inflammation," to be followed in 1875 by "The Germ Theory of Fermentative Changes," which was to bring about an even greater revolution in hospital treatment and the improved hygienic condition of the sick in hospitals. Previous to Sir Joseph Lister's investigations, and during the period in which they were being laboriously followed out to the evolution of the aseptic system, a few zealous men of various professions who were interested in hospitals had been evolving, step by step, an improved system of hospital construction, and a perfect system of hospital drainage and hygiene. Thus year by year, with slow but devoted labour, a small number of scientific men and hospital experts have brought hospital construction and the treatment of the sick in hospitals to the state of perfection which they have undoubtedly attained to-day.

The result of this has been to make a hospital in the present day one of the most popular, as it is one of the most important, elements in our social system. Formerly it was difficult to persuade a man, however serious his condition, to enter a hospital. Now, the great difficulty is to keep the majority of people who are seriously ill from seeking admission to a hospital ward. What is known as hospital abuse owes its existence, in fact, in no small degree to the confidence which everybody feels in the skill of the medical officers and the excellence of the administration of the voluntary hospitals of England. From a social point of view this is not to be wondered at, as the improvement in the education of the people and their increase in knowledge has led them to realise what is perfectly true—that for anyone who is seriously ill there is, after all, no place where recovery may be more speedily secured than in the best administered hospital. In these circumstances, it is desirable to take every opportunity of calling public attention to the wonderful development that has taken place in the hospital idea. It should not be difficult, with the co-operation of the medical profession, to make such arrangements for the admission of patients as will protect their interests and secure to all classes the full advantages which the hospitals have to offer. We cannot, however, pursue this branch of the question to-day. Those interested will find it fully discussed in the chapter on the misuse of hospitals in "Burdett's Hospitals and Charities, 1896." We propose to illustrate what we have said in a further article, which will contain a description of the most recent and interesting development of the hospital idea, *i.e.*, the Brook Hospital at Shooter's Hill.

